# Editorial. Valuing headache’s solution

**DOI:** 10.1186/s10194-021-01246-2

**Published:** 2021-06-10

**Authors:** TJ Steiner, M Tinelli

**Affiliations:** 1 Department of Neuromedicine and Movement Science, Norwegian University of Science and Technology, Edvard Griegs gate, Trondheim, Norway; 2 Department of Brain Sciences, Imperial College London, London, UK; 3 Care Policy and Evaluation Centre, The London School of Economics and Political Science, London, UK

**Keywords:** Headache, Structured headache services, Economic analysis, Value of treatment, Health policy, Global Campaign against Headache

Headache disorders are ubiquitous, common and often the cause of lifelong disability. This is so well established that it should not need repeating. Yet it does [1].

Collectively, according to the 2019 Global Burden of Disease study (GBD2019), headache disorders are the world’s third leading cause of disability, and *top* cause in young adults, responsible globally for 46.6 million years lived with disability (YLDs), 5.4 % of all YLDs [2, 3]. Because disability leads to lost productivity, headache disorders have a huge financial impact. In Europe, their total annual cost in 2012 was estimated at well in excess of €100 million [4].

Effective treatments exist for the disorders most responsible: migraine, tension-type headache and medication-overuse headache [5]. These treatments should, in a well-ordered world, substantially mitigate the losses both to health and to the world’s economies. The reality is very different. GBD2019 drew attention to headache disorders, remarking that their prominence among the ranked causes of lost health had “received little attention in global health policy debates” [2, 3]. This is true [1]. Everywhere, headache disorders are under-recognized in society and under-prioritized and under-resourced in health policy. Health-care systems that should provide these treatments either do not exist or, where they do, fail to reach many who need them [6]. Accordingly, headache disorders remain under-diagnosed and undertreated in populations everywhere [6].

While this is essentially a political failure, its causes mostly lie in education failures, occurring at all levels – political, health-care provider and general public [6]. The consequences are seen on these same levels. Health-care providers, without the requisite training or resources to manage headache effectively, achieve poor and disillusioning outcomes. People with headache who would benefit from care find services unavailable, fragmentary or difficult to access. Dissatisfied with health care that is inadequate, they fail to seek it and adhere poorly to it. Change is hard to achieve: policy makers, seeing a level of demand for care that is far below verifiable need, remain unmoved [1].

Two programmes of action have given rise to the content of this themed issue of the *Journal of Headache and Pain*.

In 2015, the European Brain Council (EBC) developed its *Value-of-Treatment* (VoT) project, building on the success of its earlier *Cost of Brain Disease* database [7]. The immediate purposes behind VoT were two-fold: first to identify barriers, stumbling blocks, pinch-points and dead-ends in the “patient’s journey through disease” (specifically, nine common neurological diseases, including headache), and second to assess the potential cost-effectiveness of interventions to ease the journey and improve care and outcomes. Overarching these was the political objective of producing evidence not only of need for change but also of the likely economic benefits of evidence-based change. Two of the manuscripts here have their origin in this project [8, 9].

Others, including three preparing the ground for economic analyses [10–12], spring from the Global Campaign against Headache. Launched in 2003 by the World Health Organization (WHO) in collaboration with the major international headache societies [13, 14], the Campaign has, since 2009, been conducted by *Lifting The Burden* (LTB), a UK-registered non-governmental organization in official relations with WHO [15, 16]. Over the years, LTB has gathered evidence from around the world of the magnitude of public ill health attributable to headache and of the inadequate responses to it [6], supporting and building research capacity in many countries while doing so [16]. It has endeavoured to use this evidence to influence policy. In particular, by informing successive GBD studies [1–3], LTB has raised political and public awareness of headache and the burdens it imposes. Finally, LTB has proposed an efficient and effective health-care solution [6, 17–19], pursuing the Campaign’s ultimate purpose. That solution is structured headache services [19].

But, if structured headache services are equitably to reach all who might benefit, the required up-front investment will be substantial. It is the task of this themed series of manuscripts to show *value* in that investment.

The content that follows, with a focus on Europe but relevant worldwide, updates the thinking behind the structured headache services model, and refines their description. With an authorship from 32 countries, it explains how these services should be organized, and how the model might be adapted for different settings [19]. It develops the methodology for economic evaluation of the model [8], including – necessary for this purpose – the introduction of a universal outcome measure applicable equally to acute and preventative treatments and to systems delivering them [10]. It applies this methodology to headache services in Europe, finding and reporting clear evidence of the model’s cost-effectiveness to justify the up-front investment in its implementation [9]. In doing this, it contributes to a better understanding, underpinned by robust empirical evidence, of the complex relationship between headache-attributed disability and lost productivity [11, 12] – a key factor in economic evaluation. Finally, it comments on the policy priorities for headache in the current context of health-systems reforms, and how we can ensure that policy, influenced by evidence built from sound research, is based on solid scientific knowledge [20].

## Data Availability

Not applicable.
